# Digital Mental Health Research Priorities, Revisited for the AI and Large Language Model Era

**DOI:** 10.2196/104118

**Published:** 2026-07-10

**Authors:** Max Valentin Birk, Shruti Kochhar, Keris Myrick, Stephen M Schueller, John Torous

**Affiliations:** 1 Eindhoven University of Technology, Eindhoven, North Brabant, The Netherlands; 2 JMIR Publications, Toronto, ON, Canada; 3 Beth Israel Deaconess Medical Center, 20 Overland St, Suite OV-202, Boston, MA, 02215, United States, 1 6176676700; 4 University of California, Irvine, Irvine, CA, United States

**Keywords:** artificial intelligence, AI, large language model, LLM, mental health, apps

## Abstract

Digital mental health has become an established part of mental health care, but the rapid arrival of large language models and other artificial intelligence (AI) tools has refocused attention on the evidence needed to guide the field. This editorial updates the research priorities articulated by *JMIR Mental Health* in 2023, while reaffirming their emphasis on equity, replicability, privacy, efficacy, and engagement. While the importance of these priorities has not changed in recent years, the urgency with which they must now be applied has. As digital tools become more clinically consequential, research must move beyond demonstrating that a technology is feasible, usable, or novel. The field now needs studies that clarify how these tools work, for whom they are beneficial, under what conditions they may cause harm, and how they can be ethically integrated into care. We call for research that is transparent about the technologies being studied, grounded in meaningful clinical questions, attentive to safety, and designed to produce knowledge that remains useful as specific products and models change.

## Introduction

Digital mental health is no longer an emerging field. Increases in teletherapy platforms and providers have increased many people’s comfort and knowledge with receiving services remotely. It might already be the case that more people are using chatbots than therapists for mental health support [1]. What started as a field based on computerized programs has expanded to include apps, virtual reality, social media, digital phenotyping, rule-based chatbots, and large language models (LLMs). Many health systems and public entities are exploring the use of digital mental health to more effectively meet people’s needs [2,3]. This shift raises the standard for the research we publish. Although establishing the feasibility of digital mental health interventions previously made a useful contribution, similar feasibility studies may fail to further advance the field. The field now needs more evidence that can guide clinical care, inform policy, protect patients, and identify which technologies work, for whom, under what conditions, and at what risk [4-6].

In 2023, *JMIR Mental Health* outlined priorities for digital mental health research centered on equity, replicability, privacy, efficacy, and engagement [7]. Those priorities remain. What has changed is the speed, scale, and consequence of the technologies now entering mental health care, especially with the emergence of LLMs. Although LLMs are the most visible example of the current methodological challenge, the standards described here apply across digital mental health. Given the recent volume of submissions on LLMs, better defining the scope of these papers will aid authors wishing to disseminate their work and provide guidance to the field on which areas of inquiry we see as most generative.

The challenges of LLMs moving faster than the research base have been well documented across the mental health field, including cases of patient harm, ongoing lawsuits, and expanding efforts to restrict or ban some tools for care. This is the bind David Collingridge [8] described decades ago: by the time evidence accumulates about a new technology, it is often too embedded in practice, regulation, or use to redirect easily. The question facing digital mental health research is no longer whether the field can produce evidence, but whether the evidence it produces is rigorous, generalizable, timely, and useful enough to inform how these technologies are evaluated and used to improve care.

**Figure 1.**
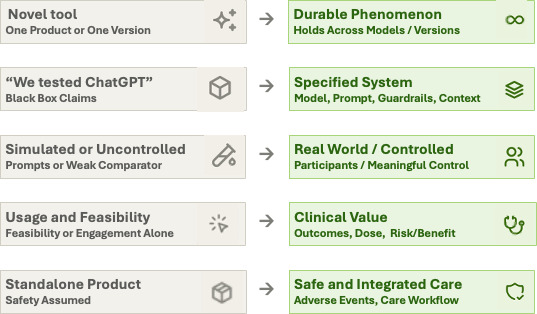
Schematic of updated research priorities, with a focus on artificial intelligence. Across all priorities, equity, safety, transparency, implementation, and clinical relevance still apply, as noted in our prior guidance.

Artificial intelligence (AI) is itself part of how that evidence is now produced. Authors today may use LLMs to draft, summarize, code, and even analyze. The volume of research on AI in mental health has risen sharply, and so has concern that the quality of that research has not kept pace. This is the paradox of the current moment: the same tools that accelerate research are also raising questions about its soundness. This is why research standards matter more than ever, and why *JMIR Mental Health* seeks to advance the most rigorous and useful research with new priorities, as summarized in Figure 1. We are seeking research that helps the field learn something durable, especially given how rapidly the field changes.

## What We Want From Research

For digital mental health work in general, and for LLM-related work in particular, the strongest studies are designed around the phenomenon being investigated rather than only the specific tool being tested, which is aligned with the National Institute of Mental Health’s experimental therapeutics approach [9]. Submissions in this space are most useful when they meet several standards.

### Durable Knowledge Beyond a Single Product

Studies should be explicit about whether they are evaluating a general phenomenon, a product, a model family, a model version, a user interface, or a deployment context. Work that examines more than one model, version, setting, or population is often better positioned to help the field draw conclusions that remain useful as technologies change. The most useful studies will identify those mechanisms or active ingredients that hold across technologies, much as the Wellcome Trust’s active ingredients commission sought to isolate the components driving benefit in mental health interventions [10] This extends the National Institute of Mental Health experimental therapeutics framing noted above [9]: even if the mechanisms underlying mental health conditions, and those of the digital tools acting on them, are never fully specified, research oriented toward mechanisms will prove more durable than research tied to a single product.

### Transparent Characterization of the Technology

Terminology and technical specificity matter more than ever as technology rapidly evolves. The base model behind a product, the wrapper or interface layer built on top of it, the system prompt, the safety guardrails, and the deployment context all shape what a study is actually measuring and can be the difference between an AI missing a safety warning or not [11]. Submissions that collapse these components into phrases such as “we tested ChatGPT” become difficult to evaluate, replicate, or interpret. Worse, they may misinform about safety, as critical protections often differ across the model, product, and API layers of technology. Reporting frameworks such as CONSORT-AI (Consolidated Standards of Reporting Trials–Artificial Intelligence), SPIRIT-AI (Standard Protocol Items: Recommendations for Interventional Trials–Artificial Intelligence), DECIDE-AI (Developmental and Exploratory Clinical Investigations of Decision Support Systems Driven by Artificial Intelligence), MI-CLAIM (Minimum Information About Clinical Artificial Intelligence Modeling), MINIMAR (Minimum Information for Medical AI Reporting), TRIPOD-AI (Transparent Reporting of a Multivariable Prediction Model for Individual Prognosis or Diagnosis–Artificial Intelligence), and AI model cards published by developers can help authors describe AI systems with better precision.

### Meaningful Comparators and Controls

The choice of comparator greatly determines what a paper can offer the field. Meaningful comparators in this space are those that control for the nonspecific effects of technology itself, including structure, attention, credibility, expectancy, and the sense of receiving a plausible intervention. Studies should be designed to isolate the active ingredient they claim to test. Recent work in this journal showed that ecologically valid online controls produced credibility, expectancy, and outcome trajectories nearly indistinguishable from purpose-built digital interventions. These controls included curated libraries of the kind of popular content that users actually encounter when seeking help online and showed a small between-condition effect size (Cohen *d*<0.15) [12]. Other recent pilot work found that an unstructured general-purpose LLM produced reductions in depression symptoms comparable to a purpose-built mental health chatbot, with both outperforming an assessment-only control [13]. Whether we call this a digital placebo effect or not, these examples demonstrate empirical data on the nonspecific effects of digital engagement.

### Clinical Outcomes and Connection to Care

The field has produced many studies showing that AI systems can respond plausibly to clinical prompts, but fewer that show these systems improve patients’ lives [14-16]. LLM mental health work has generated impressive results on simulated cases, standardized vignettes, and short interaction tasks. A recent scoping review of ChatGPT’s clinical applications in mental health found that 83% of the 60 included studies were prompt-based experiments without human participants, with only 3 controlled trials and only 6 studies enlisting clinical populations [16]. Models that perform well on benchmarks may behave less reliably in real interactions, and even models that demonstrate safe responses in single-turn exchanges may pose harms across long conversations [17]. Submissions should use measures that are practical and actionable in real-world settings and hold value for patients and clinicians, as these are most likely to shape practice. These measures move beyond symptom change to also include functional improvement, quality of life, safety, and integration into existing care pathways.

### Human Support, Safety, and Accountability

Human support should be described with the same specificity as the technology itself. Digital navigators [18], peer specialists [19,20], clinicians, and other support roles can shape engagement, safety, and outcomes, but vague claims about human oversight are not enough. At the same time, recent work has cautioned that “human-in-the-loop” oversight, when invoked without specification, can function more as symbolic reassurance than substantive protection [21]. Human-in-the-loop should not be treated as a safety claim unless the loop is described with enough detail to evaluate who is responsible, what they can see, when they act, and what authority they have to change care. Studies that include human roles should specify details such as who performs those roles, what training they have received, what data they monitor, and when they intervene.

### Reporting on Adverse Events

Clinical trials in digital mental health still lack a consensus framework for identifying and reporting adverse events, in contrast to pharmacological trials, which operate under strict regulatory oversight. Potential harms, such as worsening symptoms, distressing disengagement, and inappropriate responses to crises, are inconsistently tracked and reported across studies [22,23]. Prior efforts have proposed taxonomies of harm [24], yet these are rarely taken up in practice. AI-based interventions introduce a further complication: unlike static digital tools, the intervention itself may exhibit considerable variance, meaning that inappropriate crisis responses and problematic use require additional vigilance. Rigorous research should clearly define adverse event criteria, specify monitoring protocols, identify escalation thresholds, and report safety findings transparently. AI harms can be clear, but they can also accumulate slowly over long conversations and the formation of a dangerous parasocial relationship that may be harder to detect, yet more deadly [25].

### Theory and Data on Emerging Phenomena

New phenomena tend to outpace the literature, and that appears to be the case with AI. “AI psychosis,” while not a formal diagnostic category, has become a shorthand for concerns about delusional ideation, overreliance, reality testing, and harmful narrative reinforcement in interactions with conversational agents [26]. Theoretical contributions should do real conceptual work by locating such phenomena in relation to existing concepts, proposing mechanisms, or specifying who may be at risk and why. Empirical contributions should rest on more than illustrative examples. Ideally, new datasets can be created and shared. For phenomena where the published evidence base remains nascent, we particularly welcome detailed clinical case reports and work that includes patients as authors, whose own accounts shape the analysis rather than appearing only as data.

### Equity, Voice, and Context

The 2023 editorial called for equity in access, in research populations, and in authorship, and the years since have made all three calls more pressing. Models trained predominantly on English-language sources from high-resource areas perform less reliably in other languages and contexts, and research populations remain skewed away from the needs of low- and middle-income countries. The mismatch is highlighted in a recent conversation-level analysis [27] of ChatGPT use in India, Nigeria, Brazil, and Pakistan, which found that health-related queries occurred at substantially higher rates than in Western comparators. Equity requires asking whether the language, culture, infrastructure, costs, literacy, and care pathways assumed by a technology align with the intended user group and population. We value submissions conducted in low- and middle-income settings, work in languages other than English, studies that surface cultural variation in how digital tools are used and understood, and work that brings lived-experience perspectives into authorship.

## A Note on Scope

Much of this editorial focuses on LLMs because they raise questions for the field that are especially acute. The journal’s interests, however, remain broader, and an LLM is often not the right, or only, tool for mental health. We continue to welcome rigorous work on mental health apps, virtual and augmented reality, rule-based chatbots, digital phenotyping, online interventions, remote monitoring, digital therapeutics, social media, clinical prediction, hybrid care models, and other topics. The standards described here apply across these areas: submissions should be replicable, clinically meaningful, attentive to equity and engagement, and grounded in appropriate comparisons.

Engagement remains one of the field’s foremost challenges [28,29]. We are thus interested in work that moves beyond reporting low engagement (or perhaps in some cases, too much engagement with AI or social media) toward examining how engagement matters on a personal level. Does greater use predict better outcomes, or does it simply reflect higher baseline need? Is there a dose-response relationship, and how do we even measure dose? Should we measure minutes, sessions, completed modules, conversational turns, therapeutic content, or human contact?

With LLMs, the engagement question is now whether it is too little, as was often the case with smartphone apps. Recent passive-sensing data from over 6000 US youth found that median daily use of generative AI apps was under a minute, while a small subset engaged for more than 40 minutes a day [30]. This pattern suggests that average exposure can conceal very different individual experiences. Cases of compulsive use and parasocial attachment raise empirical questions about what too much engagement looks like, who is vulnerable to it, and what consequences may follow.

## Implementation

Implementation deserves more attention. The most clinically useful tools are not always the most technically advanced ones. Research showing how a technology fits a setting, workflow, payment model, workforce, or population is crucial. LLMs raise new challenges in implementation, design (eg, accessibility and access to data), and output (eg, bias and correctness) [31]. Real-world implementation is shaped by infrastructure, cost, training, reliability, privacy, and legal accountability as much as by underlying capability [32]. For example, recent research on the current generation of AI scribes has suggested that they may actually save negligible time, [33] and research examining the costs of running LLMs for medical coding suggests the cost may be far higher than expected [34]. Research that takes those constraints seriously rather than designing around them is especially productive.

Theories, frameworks, and models from implementation science might support structuring this research in ways to support learnings across projects. It is also worth considering the extent to which these theories, frameworks, and models apply to digital mental health and what adaptations might be necessary [35]. Given the established evidence base for many digital mental health interventions, better understanding how to successfully integrate them into routine care settings is an opportunity for future contributions.

## Real-World Evidence

As implementation increases, data generated from real-world settings has become more common. Although these investigations overcome the challenges posed by conducting clinical trials in artificial environments that do not mimic real-world deployments, they face limitations that may limit the insights they can offer the field. When used, real-world data need to be appropriate for the proposed research question, including aspects such as the size and representativeness of the sample. Information about engagement, outcome, covariates, and potential confounders is always critical. In many examples of real-world data, it may not be possible to use as rigorous a comparison of conditions as might be expected in clinical trial settings; however, this does not mean that other considerations of rigor and reproducibility are not relevant. Studies that merely examine convenience samples from deployed interventions may contain a degree of bias, and any conclusions may raise more questions than answers.

## Expanding Beyond Current Knowledge

A few categories of submission are unlikely to fit, less because they lack effort than because they no longer move the field forward without a stronger justification. Studies whose main finding is that people can use a piece of technology or find it acceptable are rarely sufficient in 2026. Cross-sectional surveys of technology use, particularly surveys measuring generic interest in AI for mental health, have been well covered. Research focused on older AI models that are no longer in use is generally of historical rather than practical interest.

Effect sizes for digital mental health interventions have also been well characterized. A meta-analysis of 176 randomized trials placed app-based effects for depression and anxiety in the small-to-modest range (Hedges *g* was approximately 0.26 to 0.28 for both depression and anxiety) [36], and a recent meta-analysis of 39 AI chatbot trials for depression and anxiety reported nearly identical estimates (Hedges *g* ranged from 0.28 to 0.31) [37]. While these effect sizes will vary based on human support, follow-up duration, clinical severity, and other factors, they at least offer a baseline to orient new findings. Papers reporting substantially larger effects are welcome, but the rigor of evidence behind them should match the size of the claim. Finally, work raising ethical concerns, including but not limited to the use of patient data without appropriate permissions, will not be considered regardless of the strength of the underlying findings.

## Transparency in AI-Assisted Research

The profound impact of generative AI tools in scholarly writing is now well known—the same tools that are shaping how digital mental health research is conducted are also driving how related scholarly work is produced [38]. These tools introduce risks spanning factual accuracy, fabricated content, hallucinated references, copyright infringement, and ethical breaches. As a result, manuscripts that do not address AI assistance at all are increasingly difficult to interpret both scientifically and ethically, challenging editors, reviewers, and readers. Authors should therefore report how and when generative AI was used in the research or writing process, specifying the tools, the nature of the AI-generated content, and, where relevant, the prompts that shaped it [39], per guidelines developed by JMIR leadership. We also appreciate and expect that many new research papers will use AI in a larger role and welcome that when fully disclosed.

## Conclusion

The potential of digital mental health has always been that technology can expand access to care while improving outcomes that matter to patients, clinicians, and communities. Today that promise is more visible, more commercially active, and more clinically consequential than ever. But promise is not evidence, and excitement is not a substitute for rigorous research, as earlier instances of enthusiasm around apps, virtual reality, and other digital tools have shown. The window Collingridge [8] described has not closed, but it is narrower than it was when *JMIR Mental Health* published its 2023 editorial guidance.

The Society of Digital Psychiatry, in partnership with the journal, has set out a roadmap for translating research into practice through education, AI standards, and digital navigators [40]. The publication standards described here are the upstream complement: research must be designed to be worth translating. Whether the next 3 years bear out the promise of digital mental health will depend less on the tools than on the evidence that shapes their use.

## Abbreviations

AI: artificial intelligence
LLM: large language model
